# Influence of Fetuin-A on *Chlamydia muridarum* Pulmonary Infection

**DOI:** 10.1155/2022/6082140

**Published:** 2022-04-19

**Authors:** Faria Mahjabeen, Jieh-Juen Yu, James P. Chambers, Rishein Gupta, Bernard P. Arulanandam

**Affiliations:** Department of Molecular Microbiology and Immunology, The University of Texas at San Antonio, San Antonio, Texas 78249, USA

## Abstract

Fetuin-A is an acute phase glycoprotein shown to counter in a regulatory manner proinflammatory cytokine production to maintain homeostasis during inflammation. We report here that in wild-type mice 12 days after *Chlamydia muridarum* (Cm) intranasal challenge, fetuin-A content in the lungs decreased 46%, while INF-*γ* increased 44%, consistent with a negative regulatory role of fetuin-A in inflammation. Importantly, the observed increased IFN-*γ* production was abrogated in fetuin-A-deficient AHSG mice suggesting that IFN-*γ* induction following Cm infection is fetuin-A dependent. Assessment of expression of genes associated with inflammation revealed fetuin-A-dependent upregulation of TBX21 (a Th1 cell-specific transcription factor) in the lungs of Cm-infected WT mice that correlated with IFN-*γ* induction. Additionally, the effect of fetuin-A deficiency in mounting an adaptive immune response to Cm infection was demonstrated using a splenocyte recall assay. Although preliminary in nature, these findings are suggestive of fetuin-A involvement following Cm pulmonary infection and underscores the need to investigate further the role of fetuin-A in the immune response and the consequences of its gene deletion.

## 1. Introduction

*Chlamydia* species are obligate intracellular bacteria and a common cause of sexually transmitted disease (STD). Our previous study showed that genital tract *Chlamydia muridarum* (Cm, adapted murine model of *Chlamydia trachomatis*) infection leads to an increased expression of fetuin-A (also known as alpha-2-HS-glycoprotein encoded by the *AHSG* gene) in C57BL/6 mice [1]. Fetuin-A, which is secreted from both the liver and adipose tissue, is found in abundance in serum and has been widely recognized as a multifunctional molecule that participates in vascular calcification, bone metabolism regulation, insulin resistance, and inflammatory response [2]. However, very little is known about the role of fetuin-A in infectious diseases. Using an *E. coli* LPS-induced endotoxemia mouse model, Li et al. reported that fetuin-A-deficient mice are more susceptible to lethal systemic inflammation [3]. Additionally, fetuin-A was found to suppress LPS-induced production of interleukin 1*β* (IL-1*β*), nitric oxide (NO), and tumor necrosis factor-alpha (TNF-*α*) in cultured macrophages, suggesting that fetuin-A is a potent anti-inflammatory acute phase protein [4].

*Chlamydia* is best known as an infectious agent for STD, and recurrent infection can lead to chronic inflammatory complications, including pelvic inflammatory disease and infertility [5, 6]. However, *Chlamydia* also causes lung infections ranging from community-acquired pneumonia, bronchitis, pharyngitis, sinusitis, chronic asthma, and obstructive pulmonary disease [7]. Initially, *Chlamydia* encounters and infects lung epithelial cells and alveolar macrophages leading to infiltration of macrophages, dendritic cells, and neutrophils [8]. Upregulation of proinflammatory cytokines and chemokines followed by CD4^+^ T cell-mediated type 1 immune response has been shown to confer protective immunity against chlamydial lung infection through NO production by iNOS pathway activation [8]. While CD8+ T cells contribute to control of bacterial growth via IFN-*γ* and TNF-*α* early in the infection, CD4+ T cells have been shown to be more important in the later stages of infection, especially in cases of reinfection [9]. To date, the influence of fetuin-A following chlamydial pulmonary infection has not been reported prompting our examining albeit in a preliminary fashion, the focus of this report.

## 2. Materials and Methods

### 2.1. Mice

C57BL/6 (WT) were purchased from Jackson Laboratory (Bar Harbor, ME). Fetuin-A knock out mice backcrossed onto C57BL/6 mice [10] (referred to as AHSG mice) were used in this study. All experimental procedures were carried out in compliance with an approved protocol issued by the Institutional Animal Care and Use Committee (IACUC) of the University of Texas at San Antonio.

### 2.2. Intranasal Infection

Seed stocks (Cm) were propagated in HeLa 229 cells. At 24 hours postinfection, cells were mechanically dislodged using glass beads, and Cm elementary bodies (EBs) were obtained using the Renografin gradient separation method as described previously [11]. The same Cm seed stock was used to infect all mice throughout the study. Mice were infected intranasally with 500 inclusion forming units (IFU) of purified seed stock suspended in 10 ul sterile sucrose-phosphate-glutamate (SPG) buffer or mock infected with only 10 ul sterile SPG buffer. At days 4 and 12, mice were euthanized, and lung and spleen tissues (day 12 only) were collected aseptically and used for downstream analysis.

### 2.3. Splenocyte and Lung Lysate Preparation

Spleen tissue single cell suspensions were prepared as previously described [12]. Lung tissue (40–100 mg) was homogenized in 1 ml SPG buffer, and resulting lysate was subjected to centrifugation (2000 rpm at 5°C for 10 minutes) to remove tissue cellular debris.

### 2.4. Bacterial Burden Assessment

Lung tissue lysate was used for IFU assessment following infection of McCoy cell monolayers and immunofluorescent staining as previously described [13, 14]. Briefly, McCoy cells were grown on sterile coverslips in 24-well plates for 24 hours, followed by infection with lung lysate diluted 1 : 80 with Eagle's minimum essential medium plus 10% fetal bovine serum (EM-10 media). The infection was terminated (18 hours postinfection) with the addition of 500 *μ*l 4% (v/v) paraformaldehyde (incubated at 5°C overnight). Cells were permeabilized with 2% (v/v) saponin, followed by blocking with EM-10, and incubated with an anti-*Chlamydia* genus-specific murine monoclonal primary antibody (1 : 1000 dilution) for 1 hour at room temperature. Goat anti-mouse IgG secondary antibody conjugated to Cy3 (1 : 100 dilution) was then added and incubated for 2 hours, followed by addition of Hoechst nuclear stain at room temperature in the dark. Coverslips were mounted on a glass slide with FluorSave^TM^ reagent (Sigma-Aldrich, St. Louis, MO) and left to dry overnight. The number of inclusion bodies was determined by immunofluorescent microscopy as previously described by Gupta and coworkers [1].

### 2.5. RNA Extraction and Quantitative Real-Time PCR

Total RNA from day 12 lung tissue (40 mg) previously immersed in liquid nitrogen and pulverized with a mortar pestle on ice was extracted using the Aurum Total RNA Mini-Kit (Bio-Rad Laboratories, Hercules, CA) and quantified (Nanodrop Spectrophotometer, ThermoScientific, Waltham, MA). RNA (2 *μ*g) exhibiting A_260/280_ values of 1.8–2.0 was converted to cDNA using the Reliance Select cDNA Synthesis Kit (Bio-Rad) with random primers as previously described by Arkatkar and coworkers [15]. The resulting cDNAs were further amplified using the SsoAdvanced PreAmp Supermix (Bio-Rad). Quantitative real-time PCR was carried out using cDNA and gene-specific primer pairs (GAPDH qMmuCED002749; HSP90ab1 qMmuCED000500; CCL3 qMmuCED0044190; HMGB1 qMmuCED0041193; SOCS1 qMmuCED0024846; TBX21 qMmuCID0022343; IL-4 qMmuCED0044969, and SMS qMmuCED0040754 (Bio-Rad)) dissolved in SsoAdvanced Universal SYBR Green Supermix (Bio-Rad), and a CFX 96 instrument was used (Bio-Rad). All mRNA expression levels were normalized to housekeeping genes GAPDH and HSP90 and reported as expression relative to that of WT naïve mouse expression using the comparative cycle threshold method [16]. Quantification of gene expression was achieved using the CFX Maestro software (Bio-Rad), and results were reported as fold-change differences as previously described [14, 15].

### 2.6. Determination of Fetuin-A Content

Lung lysate fetuin-A content was determined using the mouse fetuin-A DuoSet ELISA kit (R&D Systems, Minneapolis, MN) following manufacturer's instructions. Briefly, plates were coated with capture antibody (4 *μ*g/ml) and incubated overnight at room temperature. Plates were then washed with 1X PBS buffer containing 0.05% (v/v) Tween 20 and blocked with 1% (w/v) bovine serum albumin dissolved in 1X PBS for 1 hour at room temperature. Lung lysate or fetuin-A (0.9–60 ng/ml) standards were added to each well (100 *μ*l) and incubated for 2 hours at room temperature, followed by washing and addition of biotinylated detection antibody at a working concentration of 250 ng/ml. Following incubation for 2 hours at room temperature, streptavidin-HRP (horse radish peroxidase) was added and incubated for 20 minutes. Substrate solution (100 *μ*l 3, 3′,5, 5′-tetramethylbenzidine, BD Biosciences, Franklin Lakes, NJ) was then added, and reaction mixtures were incubated for 20 minutes at 37°C. Reactions were terminated by addition of 50 *μ*l stop solution (2.0 M H_2_SO_4_) and read at 450 and 570 nm using a Spark 10M microplate reader (Tecan, Switzerland). Following subtraction of respective 570 nm optical density readings from that at 450 nm, lung lysate fetuin-A content was determined by extrapolation to the standard curve generated for each set of samples assayed.

### 2.7. Determination of Cytokine Response

Splenocytes (2 X 10^5^/well) were incubated with either UV-inactivated Cm (1 X 10^5^/well), media (Dulbecco's modified Eagle medium plus 10% fetal bovine serum), or positive control (anti-CD3 antibody, 1 *μ*g/ml) for 72 hours at 37°C. Cells were removed by centrifugation (1200 rpm for 10 minutes at 5°C). Levels of IFN-*γ* in splenocyte supernatant material and lung tissue lysate were measured using the BD OptEIA Mouse IFN-*γ* ELISA reagents Kit (BD Bioscience) according to manufacturer's instructions.

### 2.8. Determination of Total Antibody

On day 12 postinfection, mice were cheek bled and the collected sera were analysed by ELISA. Plates were coated with approximately 1 *μ*g CPAF in sodium bicarbonate buffer (pH 9.5, 100 *μ*l), and 2-fold serially diluted serum was transferred to the respective wells. Alkaline phosphatase conjugated goat anti-mouse IgG (Southern Biotechnology Associates, Birmingham, AL) secondary antibody was added followed by washing and addition of *p*-nitrophenyl phosphate substrate (Sigma Chemical Co., St. Louis, MO). Reaction mixtures were incubated at 37°C for 1 hour, and hydrolysis of substrate was monitored at 405 nm using a Spark 10M microplate reader. Reciprocal serum dilutions were used to calculate respective total antibody titer [13].

### 2.9. Statistical Analysis

Statistical analysis was carried out using Prism 8.3 software (GraphPad, La Jolla, CA). For multiple group comparisons, two-way ANOVA with Tukey B correction was used. Statistical differences were deemed significant when *P* < 0.05.

## 3. Results

### 3.1. Fetuin-A-Deficient AHSG Mice Are Susceptible to Pulmonary *Chlamydia* Infection Comparable to WT Mice

**Figure 1:**
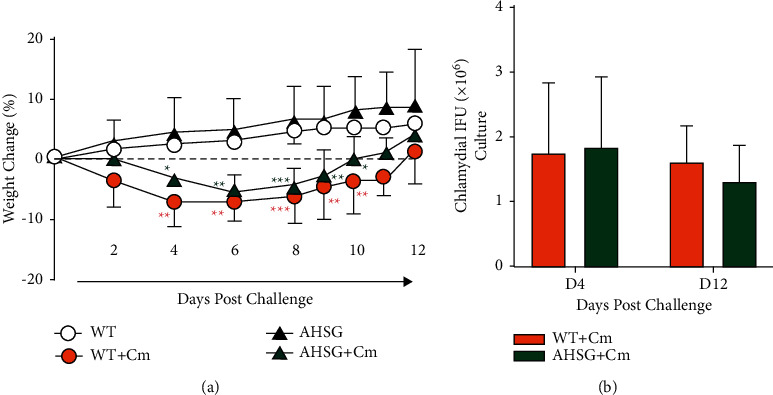
AHSG mice are susceptible to chlamydial infection. (a) Weight change as a function of days post Cm challenge. Age-matched wild-type (WT) C57BL/6 and fetuin-A-deficient AHSG mice (*n* = 6 per group) were challenged intranasally with 500 IFU Cm and monitored for weight change for 12 days. Mock (sucrose-phosphate-glutamate buffer) challenged mice were used as control. (b) Bacterial burden. At indicated days, the number of viable Cm in the lungs (*n* = 4 per group) was determined by infecting McCoy cell cultures with lung tissue homogenate for visualization of inclusions by fluorescence microscopy (cf., Materials and Methods). Weight change and *Chlamydia* IFU data represent the mean ± SD. Differences in weight change between the same strain of Cm-infected and mock-treated mice were analysed by 2-way ANOVA with Tukey's multiple comparison test. ^*∗*^*P* < 0.05, ^*∗∗*^*P* < 0.01, ^*∗∗∗*^*P* < 0.001.

Previously, we observed reduction of fetuin-A in the Cm-infected mouse genital tract [1]. However, *Chlamydia* is not only a causative agent of sexually transmitted disease but also contributes to 16–22% of all reported cases of pneumonia in the U.S. occurring within 2 months after birth. We have established a murine intranasal chlamydial challenge model to study the protective role of IFN-*γ* in pulmonary Cm infection [14, 17, 18]. Using this animal model, we assessed the role of fetuin-A in pulmonary Cm infection. Specifically, age-matched (6–8 weeks old) WT and fetuin-A-deficient AHSG mice were intranasally challenged with a sublethal dose (500 IFU) of Cm. As shown in Figure 1(a), WT mice exhibited significant weight loss as early as 4 days post Cm challenge, but gradually recovered having fully regained weight lost by day 12. Similarly, AHSG mice experienced significant weight loss at day 4 but regained weight lost by day 11 post Cm challenge. However, there was no difference in weight loss between Cm-infected WT and AHSG mice at any observed time point. Lung bacterial burden was also comparable between WT and AHSG mice at days 4 and 12 post Cm challenge. Though not statistically significant, viable Cm decreased from day 4 to day 12 in the lungs of both strains of mice.

### 3.2. Pulmonary *Chlamydia* Infection Reduced Fetuin-A Content Which Is Associated with Increased IFN-*γ* in the Lungs

**Figure 2:**
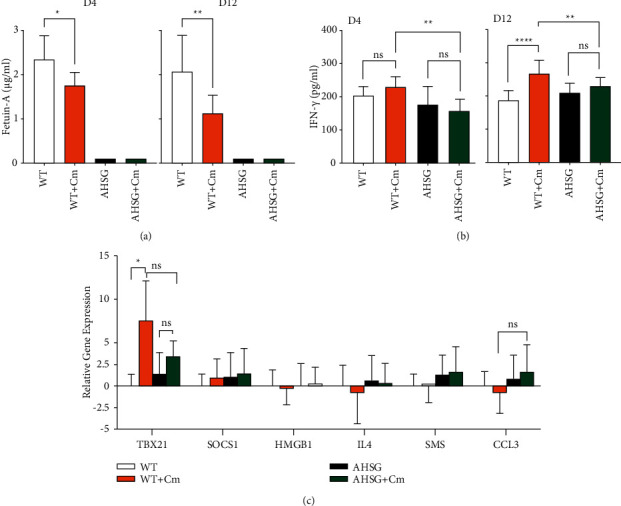
Fetuin-A content is reduced following intranasal *Chlamydia* challenge and is associated with increased IFN-*γ* content in the lung. Age-matched wild-type (WT) C57BL/6 and fetuin-A-deficient AHSG mice were challenged intranasally with 500 IFU Cm. Lung tissue (days 4 and 12) was collected and homogenized as described under “Materials and Methods.” (a) Fetuin-A content. (b) IFN-*γ* content. (c) Relative expression (fold-change) of TBX21, SOCS1, HMGB1, IL-4, SMS, and CCL3 genes. Determination of fetuin-A and IFN-*γ* content and real-time quantitative reverse transcription analysis were carried out as described under “Materials and Methods.” Respective bars represent the mean ± SD. Differences between indicated groups were analysed by one-way ANOVA with Tukey's multiple comparison test. ^*∗*^*P* < 0.05, ^*∗*^^*∗*^*p* < 0.01, ^*∗∗∗*^*p* < 0.001, and ^*∗∗∗∗*^*p* < 0.0001.

To the best of our knowledge there is no previously reported experimental animal study detailing the role of fetuin-A in bacterial infection besides that of genital *Chlamydia* [1]. Interaction(s) between host and pathogen during infection are complex. Types of immune cells activated and effector kinetics, as well as sites and number of pathogens inoculated, all contribute to the outcome*, i.e.,* disease. Pulmonary Cm infection as described in this study did not result in significant differences in animal weight loss or bacterial burden between WT and AHSG mice (Figure 1). However, pulmonary Cm infection significantly reduced lung fetuin-A content at day 4 (from 2.35 *μ*g/ml to 1.77 *μ*g/ml) after challenge in WT mice (Figure 2(a)). As expected, no fetuin-A was detected in AHSG mice with or without Cm infection. Fetuin-A content was further reduced in Cm-infected WT mice at day 12 (from 2.08 *μ*g/ml to 1.12 *μ*g/ml; Figure 2(a)) accompanied with significantly increased IFN-*γ* production (from 188 pg/ml to 271 pg/ml; Figure 2(b)). This IFN-*γ* upregulation was abrogated in AHSG mice suggesting fetuin-A-dependent IFN-*γ* induction following pulmonary Cm infection. We further assessed the role of fetuin-A in regulating other immune molecules in the lung at day 12 after Cm challenge by qRT-PCR analysis. Genes analysed included previously reported immune molecules associated with fetuin-A regulation or pulmonary Cm infection, i.e., SOCS1 [19], HMGB1 [3,20], IL-4 [21], and CCL3 [21] as well as TBX21 (a Th1 cell-specific transcription factor) that regulates IFN-*γ* expression [22]. As shown in Figure 2(c), TBX21 gene expression was markedly increased, i.e., induced following Cm infection in the WT mice, but not in AHSG mice. Furthermore, TBX21 gene expression in Cm-infected AHSG mice was significantly lower than in infected WT mice. Thus, Cm-induced AHSG-dependent TBX21 expression changes correlated with IFN-*γ* protein content (Figure 2(b), D12). In contrast to TBX21, expression of the CCL3 (a proinflammatory chemokine) gene was higher in Cm-infected AHSG mice compared to infected WT mice (Figure 2(c)). However, unlike TBX21, the dependence of CCL3 expression on fetuin-A in response to pulmonary Cm challenge could not be established. Differential expression of SOCS1, HMGB1, IL-4, and SMS genes was not observed in either WT and AHSG mice following Cm challenge.

### 3.3. The Absence of Fetuin-A Affects the Adaptive Immune Response

**Figure 3:**
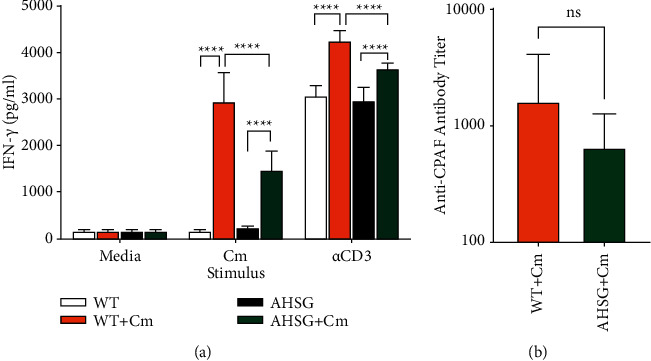
Regulatory role of fetuin-A in adaptive immunity following pulmonary *Chlamydia* infection. Age-matched wild-type (WT) C57BL/6 and fetuin-A-deficient AHSG mice were challenged intranasally with 500 IFU Cm. (a) Splenocyte IFN-*γ* secretion (*n* = 6 per group) from day 12 after Cm challenged animals stimulated with medium alone, UV-inactivated Cm, or anti-CD3 antibody. IFN-*γ* was determined 72 hours poststimulation. (b) Antichlamydial protease-like activity factor (CPAF) elicited titers. Serum (*n* = 6 per group) was collected at day 12 after Cm challenge. Preparation of splenocytes, IFN-*γ* quantitation by sandwich ELISA, collection of serum, and determination of CPAF titer were carried out as described under “Materials and Methods.” Respective bars represent the mean ± SD. Differences between indicated groups were analysed by one-way ANOVA with Tukey's multiple comparison test. ^*∗∗∗∗*^*P* < 0.0001. ns, differences not statistically significant as determined by the two-tailed Student' *t*-test.

The influence of fetuin-A on Cm infection induced cell-mediated immunity, and humoral response was assessed by splenocyte recall and antibody titer against Cm antigens, respectively. Single spleen cell suspensions prepared from uninfected and Cm-infected mice were stimulated with UV-inactivated Cm, medium (negative control), and anti-CD3 antibody (positive control) for 72 hours, and induction of IFN-*γ* was measured. As shown in Figure 3(a), all splenocytes produced minimal IFN-*γ* when incubated with media alone, but reacted strongly to anti-CD3 antibody stimulation, indicating the T cells prepared from either WT or AHSG mice are functional and can be activated via T cell receptor (TCR) signaling. As expected, Cm-primed splenocytes from WT mice produced high levels of IFN-*γ* (2.94 ng/ml) upon Cm stimulation, while unprimed cells secreted minimal amounts of the cytokine (Figure 3(a)). Similarly, primed splenocytes from AHSG mice produced IFN-*γ*, but at a significantly lower level (1.46 ng/ml) compared to WT cells when recalled with UV-inactivated Cm (Figure 3(a)). Furthermore, infection of AHSG mice with Cm did not induce significant amount of IFN-*γ* in the lungs (Figure 2(b)) suggesting fetuin-A deficiency might reduce recruitment of IFN-*γ*-secreting immune cells to the infection site and/or weaken the immune cell's effector function as evident by the splenocyte recall assay. These recall data support the contribution of fetuin-A in the mounting of the host cell-mediated immune response to Cm infection. However, the humoral response to Cm infection was less affected by the absence of fetuin-A. As shown in Figure 3(b), antibody titer elicited to chlamydial protease-like activity factor (CPAF, a dominant *Chlamydia* antigen) in AHSG mice was lower but not statistically significant when compared to WT mice at day 12 post Cm challenge.

## 4. Discussion

Although a wide range of biological functions have been proposed for fetuin-A based on its structural similarities to other proteins and/or physical interactions, fetuin-A function remains poorly understood in infectious disease. Data obtained from this study are the first to reveal an immune regulatory role for fetuin-A following Cm infection. Specifically, pulmonary Cm infection suppresses expression of fetuin-A (an anti-inflammation regulator) leading to increased IFN-*γ* production via increased TBX21 expression. Although fetuin-A deficiency did not affect weight loss and bacterial burden following sublethal Cm challenge in this study, the pathogenic and/or protective role(s) in lethally Cm challenged mice and other bacterial caused diseases remain to be elucidated.

Li et al. observed IFN-*γ* decreased basal level expression of fetuin-A in HepG2 cells [3]. However, our data (Figure 2(a) and 2(b)) suggest fetuin-A may suppress IFN-*γ*. This fetuin-A/IFN-*γ* feedback loop could influence inflammation status at the infection site and play an important role in disease progression. Furthermore, Li et al. [3] also observed that supplementation with exogenous fetuin-A inhibited release of HMGB1, a proinflammatory cytokine inducer [23] in LPS-stimulated macrophages. However, we did not observe a discernible increase in expression of HMGB1 in Cm-infected WT lung lysate compared to that of AHSG animals at day 12 when there is a significant difference in fetuin-A expression in the two mice strains. Data reported here indicate an increase in TBX21 expression, but no discernible change in SOCS1 (an IFN-*γ* signaling suppressor [24, 25]) and IL-4 (Th-2 promoting cytokine) expression levels 12 days postinfection. Increased relative expression of TBX21 in Cm-infected WT animals could be in response to observed increased levels of IFN-*γ* in a fetuin-A-dependent manner that also impacts cell-mediated adaptive immunity in response to Cm infection. Lack of fetuin-A-dependent IL-4 expression appears to be associated with the minor difference in the generation of anti-CPAF antibody between WT and AHSG mice (Figure 3(b)). However, the impact of fetuin-A on host humoral immune response to Cm infection requires further investigation beyond that of the single antigen tested in this study. Although preliminary in nature, findings presented here underscore the need to further investigate the regulatory role of fetuin-A in Cm-induced inflammation using WT and AHSG mice, e.g., determination of type and kinetics of immune cell recruitment to the infection site and regulation of immune gene activation in Cm-infected primary cells.

## Data Availability

The data used to support the findings of this study are available from the corresponding authors upon request.
